# Supplementary material for: Impact of Different Temperatures on Activity of the Pest Monolepta hieroglyphica Motschulsky (Coleoptera: Chrysomelidae)
Source: Insects. 2025 Feb 18;16(2):222. doi: 10.3390/insects16020222 (PMC11856831; doi:10.3390/insects16020222)
Supplement: Supplementary file 1 [file insects-16-00222-s001.zip › insects-3427054-supplementary.docx]

**Supplemental Figure S1**: Standard curve of antioxidant enzyme activity levels


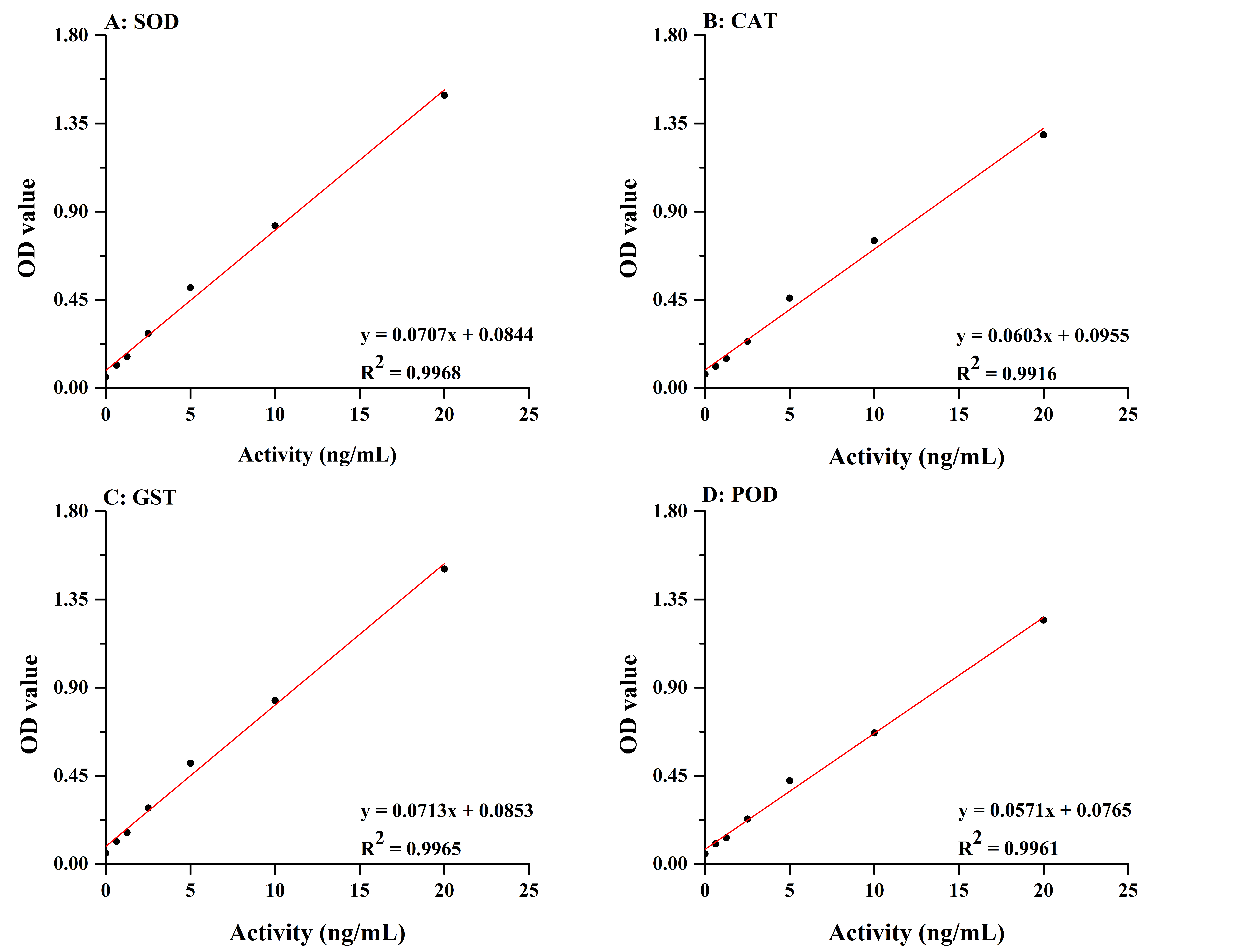


**Supplemental Table S1.** Effects of temperature stress on the detoxification enzyme activity levels of adult *Monolepta hieroglyphica* under different extension of treatment time.

| **Species** | **Time (h)** | **Temperature (℃)** | | | | **Temperature** | | **Time** | |
| --- | --- | --- | --- | --- | --- | --- | --- | --- | --- |
|  |  | **25** | **28** | **31** | **34** | ***F*** | ***p*** | ***F*** | ***p*** |
| SOD activity | 24 | 3.11±0.08Aa | 3.15±0.05Aa | 3.44±0.03Ab | 3.57±0.06Ab | 51.14 | 0.000 | 1.48 | 0.301 |
|  | 48 | 3.12±0.07Aa | 3.20±0.09Aa | 3.47±0.08Ab | 3.62±0.03Ab |  |  |  |  |
|  | 72 | 3.09±0.08Aa | 3.16±0.04Aa | 3.52±0.02Ab | 3.80±0.01Bc |  |  |  |  |
| CAT activity | 24 | 4.39±0.04Aa | 4.47±0.02Aab | 4.68±0.04Ab | 5.10±0.05Ac | 38.61 | 0.000 | 2.44 | 0.168 |
|  | 48 | 4.36±0.03Aa | 4.47±0.05Aa | 4.72±0.04Ab | 5.22±0.09Ac |  |  |  |  |
|  | 72 | 4.39±0.03Aa | 4.43±0.07Aa | 4.99±0.07Bb | 5.49±0.07Bc |  |  |  |  |
| GST activity | 24 | 3.11±0.04Ac | 3.09±0.02Ac | 2.54±0.05Ab | 1.88±0.07Ba | 38.19 | 0.000 | 1.18 | 0.369 |
|  | 48 | 3.12±0.05Ac | 3.12±0.03Ac | 2.56±0.03Ab | 1.80±0.03Ba |  |  |  |  |
|  | 72 | 3.10±0.02Ac | 3.10±0.07Ac | 2.52±0.08Ab | 1.14±0.02Aa |  |  |  |  |
| POD activity | 24 | 4.18±0.03Bb | 4.19±0.05ABb | 4.47±0.02Ac | 3.86±0.04Ca | 11.62 | 0.007 | 0.78 | 0.501 |
|  | 48 | 4.20±0.05Bb | 4.05±0.02Ab | 4.68±0.06Bc | 3.32±0.06Ba |  |  |  |  |
|  | 72 | 3.92±0.04Ab | 4.26±0.07Bc | 4.64±0.08ABd | 2.98±0.07Aa |  |  |  |  |

Different letters indicate statistically significant difference at *p* < 0.05 (ANOVA followed by a Tukey’s post hoc test). Capital letters indicate differences between different processing times at the same temperature; Lowercase letters indicate the differences between different processing temperatures under the same processing time.
